# JNK1/2 Activation by an Extract from the Roots of *Morus alba* L. Reduces the Viability of Multidrug-Resistant MCF-7/Dox Cells by Inhibiting YB-1-Dependent MDR1 Expression

**DOI:** 10.1155/2013/741985

**Published:** 2013-07-24

**Authors:** Youn Kyung Choi, Sung-Gook Cho, Hyeong Sim Choi, Sang-Mi Woo, Yee Jin Yun, Yong Cheol Shin, Seong-Gyu Ko

**Affiliations:** Laboratory of Clinical Biology and Pharmacogenomics, Department of Preventive Medicine, College of Korean Medicine, Kyung Hee University, 1 Hoegi, Dongdaemun, Seoul 130-701, Republic of Korea

## Abstract

Cancer cells acquire anticancer drug resistance during chemotherapy, which aggravates cancer disease. MDR1 encoded from multidrug resistance gene 1 mainly causes multidrug resistance phenotypes of different cancer cells. In this study, we demonstrate that JNK1/2 activation by an extract from the root of *Morus alba* L. (White mulberry) reduces doxorubicin-resistant MCF-7/Dox cell viability by inhibiting YB-1 regulation of *MDR1* gene expression. When MCF-7 or MCF-7/Dox cells, where MDR1 is highly expressed were treated with an extract from roots or leaves of *Morus alba* L., respectively, the root extract from the mulberry (REM) but not the leaf extract (LEM) reduced cell viabilities of both MCF-7 and MCF-7/Dox cells, which was enhanced by cotreatment with doxorubicin. REM but not LEM further inhibited YB-1 nuclear translocation and its regulation of *MDR1* gene expression. Moreover, REM promoted phosphorylation of c-Jun NH2-terminal kinase 1/2 (JNK1/2) and JNK1/2 inhibitor, SP600125 and rescued REM inhibition of both MDR1 expression and viabilities in MCF-7/Dox cells. Consistently, overexpression of JNK1, c-Jun, or c-Fos inhibited YB-1-dependent MDR1 expression and reduced viabilities in MCF-7/Dox cells. In conclusion, our data indicate that REM-activated JNK-cJun/c-Fos pathway decreases the viability of MCF-7/Dox cells by inhibiting YB-1-dependent *MDR1* gene expression. Thus, we suggest that REM may be useful for treating multidrug-resistant cancer cells.

## 1. Introduction

MDR1 (also called P-glycoprotein or ABCB1) encoded from a multidrug-resistant gene, *MDR1 *(or *ABCB1*), mainly mediates multidrug resistance by an efflux of drugs [1–4]. Therapeutic approaches for treating cancer in clinics are hampered by MDR1-induced multidrug resistance [3–8]. Multidrug resistance of cancer cells acquired by MDR1 expression involves a transcriptional activity of Y-box binding protein 1 (YB-1) [9–15]. Doxorubicin-resistant MCF-7/Dox cells were constructed by consecutive treatment of doxorubicin (or adriamycin) [1]. This cell line highly expressing MDR1 is resistant to various anticancer drugs including doxorubicin, paclitaxel, vincristine, and etoposide, thereby being used widely for deciphering multidrug-resistant mechanisms *in vitro* [16]. 

It has been revealed that c-Jun NH2-terminal kinase 1/2 (JNK1/2) regulates MDR1 expression via c-Jun in multidrug-resistant gastric and pancreatic cell lines [17]. Likewise, JNK1/2 mediated hypoxia-induced MDR1 expression in HOP62 nonsmall lung cell carcinoma cell line [18]. In addition, AP-1 negatively regulated YB-1-mediated *MDR1* gene expression in MCF-7/Dox cell line [19]. In MCF-7 cells, MDR1 promoter activity was also negatively regulated by c-Fos [20]. Those findings suggest that JNK1/2-mediated signaling inhibits YB-1-dependent *MDR1* gene expression and causes a loss of multidrug-resistant phenotype to anticancer drugs. Furthermore, it is recently found that MDR1 silencing reduced the proliferation of multidrug-resistant cancer cells [21]. Therefore, while the inhibition of MDR1 channel function allows chemotherapeutic agents to be accumulated in the cells, the suppression of MDR1 expression itself is also likely to be enough to attenuate multidrug-resistant cancer cell growth.

Parts of *Morus alba *L. (white mulberry) including roots and leaves have been widely used in the traditional medicine for curing symptoms such as diabetes, edema, eczema, anemia, bleeding, dry constipation, fever, sore throat, headache, muscle aches and pain, and itching [22]. Recently, extracts from *Morus alba *L. have been revealed to affect cancer disease. REM caused apoptotic cell death of different types of cancer cells such as K562 and B380 human leukemia cells and B16 mouse melanoma cells [23]. Albanol A isolated from REM also induced apoptotic cell death of human leukemic HL-60 cells [24]. Likewise, LEM inhibited neuroblastoma cells [25]. 2-Arylbenzofuran derivatives isolated from LEM also showed cytotoxicity on different cancer cells: A549 (human lung cancer cells), BEL7402 (human liver cancer cells), BGC823 (human gastric cancer cells), HCT8 (human colon cancer cells), and A2780 (human oophoroma cells) [26]. Chalcone derivatives from LEM also showed cytotoxicity in HCT-8 and BGC823 [27]. In addition, lectin purified from LEM caused apoptotic cell death of both MCF-7 breast cancer cells and HCT-15 human colon cancer cells [28, 29]. Thus, REM, LEM, and their chemical components appear to have anticancer effects. However, it is unclear whether those have anticancer effect even in multidrug-resistant cancer cells. 

In this study, we examined whether REM or LEM affects drug-resistant cancer cells. Our data present here that REM but not LEM reduces the viability of MCF-7/Dox cells highly expressing MDR1. This REM effect was due to JNK1/2 inhibition of YB-1-dependent MDR1 expression in multidrug-resistant cells. Thus, our present study provides knowledge for a role of REM against drug-resistant cancers. 

## 2. Materials and Methods

*Extract preparation, chemicals, and cell culture.* Extracts from *Morus alba* L. (REM and LEM) were prepared by and obtained from Hanpoong Pharmaceutical Company (Jeonju, Korea) following the good manufacturing practices (GMP) procedures. In brief, herbs were boiled with 80% ethanol at 100°C, and filtered extracts were then concentrated and dried by vacuum at 60°C. The dried powers were lyophilized and then dissolved in distilled water. To qualify REM and LEM, HPLC analyses were performed by Hanpoong Pharmaceutical Company (Jeonju, Korea). MCF-7 and MCF-7/Dox cells were routinely cultured in DMEM with 10% fetal bovine serum and 1% antibiotics. For transient transfections, cells were transfected with mixtures of DNAs with Lipofectamine 2000 reagents (Invitrogen). SP600125, SB203580, PD98059, and LY294002 were obtained from Sigma. 

*Cell viability.* Cells were cultured in 96-well plates and subjected to the Cell Proliferation assays (Promega). Cells were treated with the extracts for 72 hours and then subjected to the assays. All experiments were performed in triplicate. Data were represented by mean ± standard deviation. *P* values lower than 0.05 in Student's *t*-tests were considered statistically significant. 

*RT-PCR, Western Blot and Immunocytochemistry.* Total RNAs were extracted with TRIzol (Invitrogen). Syntheses of cDNA were routinely performed by MMLV reverse transcriptase and random primers. PCR to detect *MDR1* mRNA was then performed. *GAPDH* was used for an internal control. Primers used are as follows: 5′-AATCCCATCACCATCTTCCA-3′ (*GAPDH* forward primer) and 5′-TGGACTCCACGACGTACTCA-3′ (*GAPDH* reverse primer). Protein was obtained by cell lysis with RIPA buffer, and total 30 *μ*g of protein was loaded onto 6 to 10% SDS-PAGEs. Antibodies for pAKT, AKT, pERK1/2, ERK1, pJNK1/2, JNK1, pp38MAPK, p38MAPK, YB-1, and MDR1 were obtained from Cell Signaling. As an internal control, *α*-tubulin or actin was used. For the immunoprecipitation assays, anti-YB-1 or anti-JNK1/2 antibody was used. 50 *μ*L of protein A/G bead slurry and 2 *μ*g of the appropriate antibody were mixed with 200 *μ*g of protein. 30 *μ*g of protein was loaded as an input. For the nuclear fractionation, cells were lszed with buffer A (10 mM HEPES, 1.5 mM MgCl_2_, 10 mM KCl, 0.5 mM DTT, and 0.05% NP-40), and then the supernatants were used for the cytosolic fractions. The pellets were mixed with buffer B (5 mM HEPES, 1.5 mM MgCl_2_, 0.2 mM EDTA, 0.5 mM DTT, and 26% glycerol) and 300 mM NaCl and homogenized with 20 full strokes in Dounce homogenizer on ice. After centrifugation, the supernatants were used for the nuclear fractions. For the immunocytochemistry, cells were fixed with 4% formaldehyde, permeabilized with 0.5% Triton X-100, incubated in phosphate buffered saline containing 10% FBS, and then incubated with the anti-YB-1 antibody (Cell Signaling) and Alexa Fluor-488 goat anti-rabbit IgG antibody (Invitrogen). For the counter staining, phalloidin (Sigma) and TOPRO-3 (Invitrogen) were used to detect actin (especially, F-actin) and the nucleus. The object was 20x, and scale bars in the image indicate 50 *μ*m. 

*Rhodamine 123 Efflux Assay. *Cells were treated with the extracts for 30 hours and incubated for another 1 hour with 1 *μ*g/mL of the rhodamine 123. Accumulation of Rhodamine 123 in cells was analyzed by flow cytometry.

*Chromatin Immunoprecipitation (ChIP) Assays*. To analyze YB1 interaction with MDR1 promoter region, we performed ChIP assays according to the manufacturer's procedures (Abcam). Briefly, nuclear fractions from the cells fixed with 1% formaldehyde were sonicated, and then anti-YB-1 antibody (Cell Signaling) was used for chromatin immunoprecipitation. For the positive control, anti-histone H3 antibody provided from the manufacturer (Abcam) was used. For the negative control, bead-only samples were used according to the manufacturer's procedure. YB-1-bound DNAs were then amplified with primers for MDR1 promoter region and *GAPDH* primers as the internal control. Data were obtained by normalizing ddCT from real-time PCR. The values indicate the mean ± standard deviation from the experiments done in triplicate. *P*-value below 0.05 was considered statistically significant. ChIP primers used are as follows: YB-1 binding sites on MDR1 promoter, 5′-CAGTAGTGAAGCTGTAGGAC-3′, 5′-ATCAGAACTTGCTGTTCTGC-3′, GAPDH 5′-AATCCCATCACCATCTTCCA-3′, and 5′-TGGACTCCACGACGTACTCA-3′.

*Luciferase Assays.* Cells were transfected with MDR1-luc plasmid (pMDR1-1202, Addgene plasmid 37627) [30] and subjected to the luciferase assays (Promega). Extracts were treated for 6 hours. All experiments were performed in triplicate, and Student's *t*-test was performed to determine statistics. *P* value below 0.05 was considered statistically significant. All data were represented as the mean ± standard deviation.

## 3. Results 

### 3.1. REM but Not LEM Reduces Cell Viabilities of MCF-7 and MCF-7/Dox

**Figure 1:**
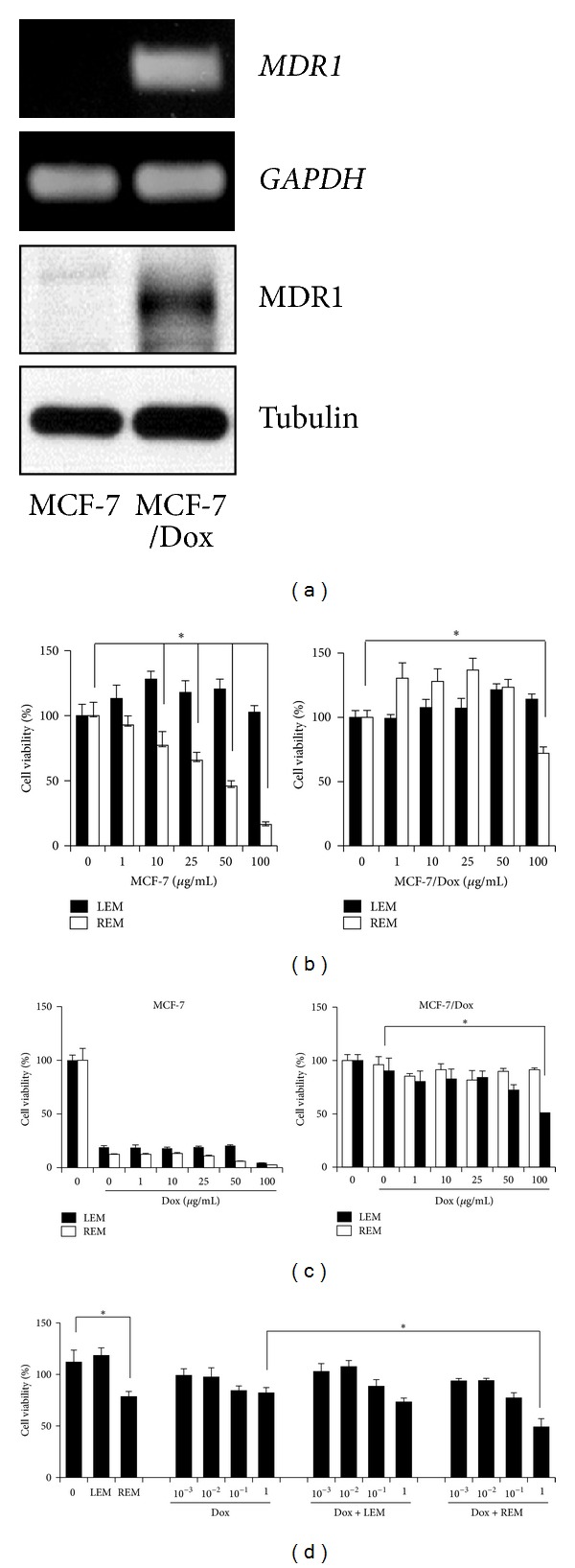
REM reduces cell viabilities of MCF-7/Dox cells. (a) MDR1 mRNA and protein levels were examined in MCF-7 and MCF-7/Dox cells. *GAPDH* and tubulin were used as internal controls. (b–d) MCF-7 and MCF-7/Dox cells were treated with the indicatives for 72 hours and then subjected to the MTT assays. The experiments were performed in triplicate, and *P*-value less than 0.05 (marked with asterisks, ∗) was considered statistically significant.

We first examined both mRNA and protein levels of MDR1, a key mediator of multidrug-resistant phenotype, in MCF-7 and MCF-7/Dox cells. MCF-7/Dox cells resistant to doxorubicin expressed MDR1 mRNA and protein, while MCF-7 cells did not (Figure 1(a)). Thus, we next examined whether our herbal extracts, REM and LEM, affect viabilities of MCF-7 and MCF-7/Dox cells. REM but not LEM reduced MCF-7 cell viability in a dose-dependent manner (Figure 1(b), left). In addition, REM at 100 *μ*g/mL also decreased MCF-7/Dox cell viability by approximately 30% (Figure 1(b), right). 

Thus, we further examined whether a combinatorial treatment of doxorubicin with REM or LEM causes a decrease of cell viability. Doxorubicin (Dox) alone strongly reduced the viability of MCF-7 cells, and its combination with various concentrations of REM or LEM appeared to more reduce it when higher concentrations of REM or LEM was combined (Figure 1(c), left). In MCF-7/Dox cells, REM at 100 *μ*g/mL, when combined with 1 *μ*g/mL of doxorubicin, decreased the viability by approximately 50% (Figure 1(c), right). However, we could not observe any reduction of multidrug-resistant cell viability in a combined treatment of doxorubicin with LEM. Subsequently, the MCF-7/DOX cells were treated with various concentrations of doxorubicin (10^−3^ to 1 *μ*g/mL) in the presence or absence of either 100 *μ*g/mL of REM or LEM. When combined with doxorubicin at 1 *μ*g/mL, REM at 100 *μ*g/mL but not LEM more reduced the viability by approximately 27%. 

### 3.2. REM-Induced JNK1/2 Activation Inhibits MDR1 Expression

**Figure 2:**
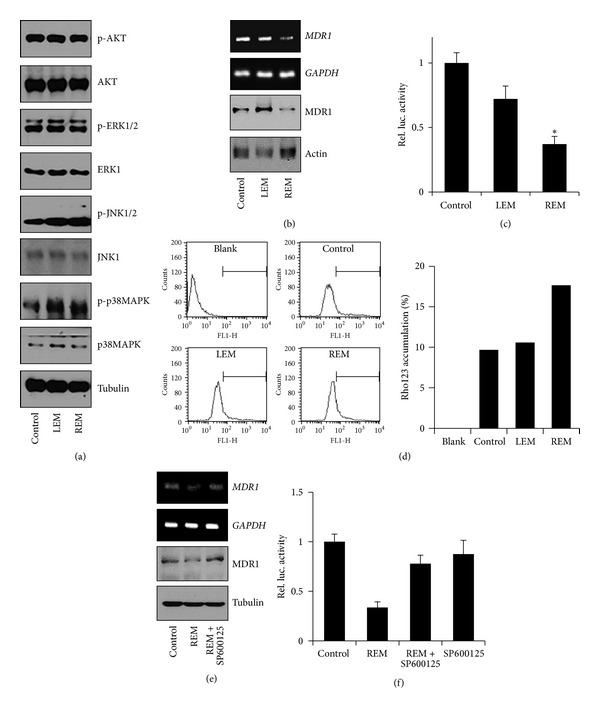
REM-induced JNK1/2 activation inhibits MDR1 expression. (a) Cells were treated with 100 *μ*g/mL of either LEM or REM for 15 minutes. Tubulin was detected as an internal control. (b) Cells were treated with the indicatives for 24 hours, and then MDR1 mRNA and protein levels were examined. *GAPDH* and actin were used as internal controls. (c) To analyze the MDR1 promoter activities, MCF-7/Dox cells were transfected with MDR1-luc construct for 24 hours and then treated with the indicatives for another 6 hours. The luciferase assays were performed in triplicate, and *P* value less than 0.05 (marked with an asterisk, ∗) was considered statistically significant. (d) MCF-7/Dox cells were treated with the indicatives for 30 hours and then incubated with rhodamine 123 for another 1 hour. Rhodamine 123 accumulation rate was analyzed by flow cytometry. (e) Cells were pretreated with SP600125 for 30 minutes and then treated with 100 *μ*g/mL of REM. 24 hours after treatment, MDR1 mRNA and protein levels were examined. *GAPDH* and tubulin were used as internal controls. (f) To examine MDR1 promoter activities, MCF-7/Dox cells were transfected with MDR1-luc construct for 24 hours and then treated with REM for another 6 hours. SP600125 was treated 30 minutes before REM treatment. The luciferase assays were performed in triplicate and *P*-value less than 0.05 (marked with an asterisk, ∗) was considered statistically significant.

To decipher REM-mediated intracellular signaling pathways on MCF-7/Dox cells, MAPKs, and AKT, proteins known for cell proliferation and survival were examined. While REM and LEM did not alter phosphorylation of AKT and ERK1/2 in MCF-7/Dox cells, both extracts increased phosphorylation of p38MAPK. Furthermore, REM but not LEM increased JNK1/2 phosphorylation of (Figure 2(a)). Thus, REM is likely to selectively regulate JNK1/2 phosphorylation. 

As JNK1/2 has been revealed to regulate MDR1 expression [17], we next examined whether REM affects MDR1 expression in MCF-7/Dox cells via JNK1/2. When MCF-7/Dox cells were treated with 100 *μ*g/mL of either REM or LEM for 24 hours, REM but not LEM reduced mRNA and protein levels of MDR1 (Figure 2(b)). Thus, we further examined whether REM affects MDR1 expression in a transcription level. MCF-7/Dox cells were transfected with MDR1-luc construct and then treated with REM or LEM for 6 hours. While LEM did not affect MDR1 promoter-mediated luciferase activity, REM reduced it by approximately 70% (Figure 2(c)). In addition, REM but not LEM increased accumulation rate of rhodamine 123 in the cells (Figure 2(d)). 

When MCF-7/Dox cells were pretreated with JNK1/2 inhibitor, SP600125, prior to REM treatment, REM inhibition of MDR1 expression was rescued by JNK1/2 inhibition (Figure 2(d)). Furthermore, JNK1/2 inhibition also rescued REM inhibition of MDR1 promoter activity (Figure 2(e)), indicating that REM-mediated JNK1/2 activation is likely important for the inhibition of MDR1 expression. 

### 3.3. REM Inhibits YB1-Dependent MDR1 Expression

**Figure 3:**
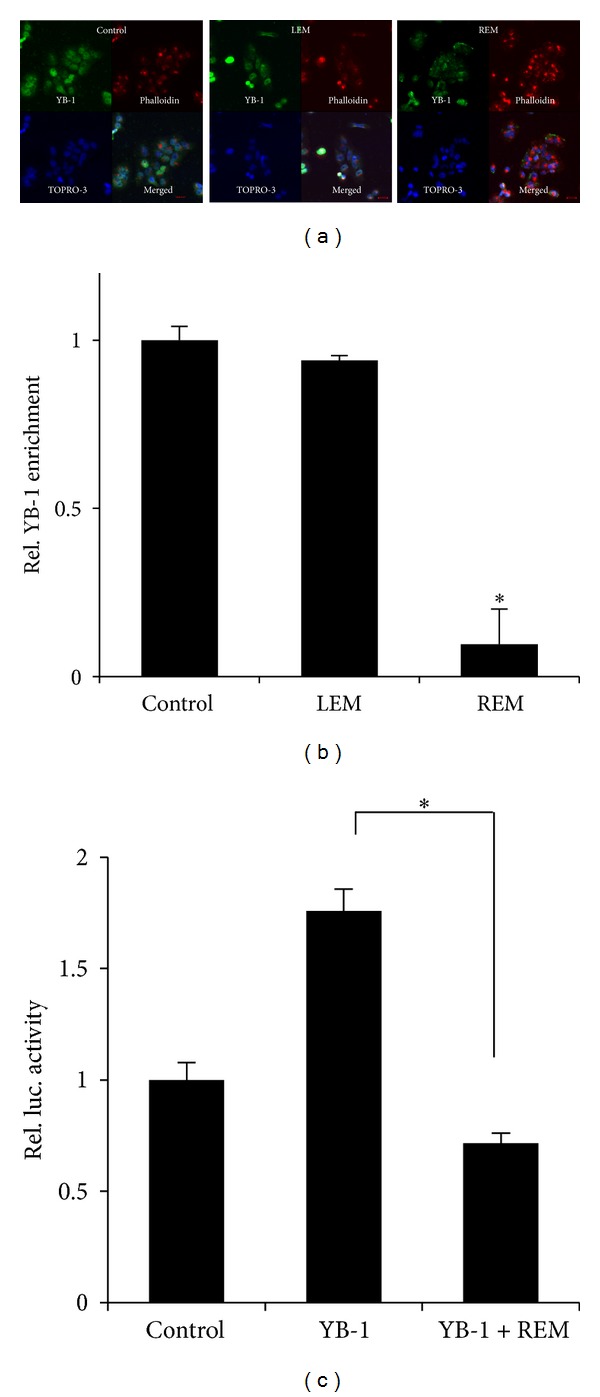
REM inhibits YB-1-dependent MDR1 expression. (a) YB-1 localization. Cells were stained with anti-YB-1 antibody (green), phalloidin (red), and TOPRO-3 (blue) to visualize YB-1 intracellular localization, actin, and nucleus. Scale bars, 50 *μ*m. The objective, 20X. (b) MCF-7/Dox cells were treated with the indicatives for 6 hours and then fixed with 1% formaldehyde. Fragmented DNAs were incubated with the appropriate antibodies, and then PCR was routinely performed. *GAPDH* was used as an internal control. Relative values of YB-1 enrichment normalized by ddCT were obtained by experiments performed in triplicate. *P*-value less than 0.05 (marked with an asterisk, ∗) was considered statistically significant. (c) MCF-7/Dox cells were cotransfected with MDR1-luc and YB-1 constructs for 24 hours and then treated with the indicatives for another 6 hours. Assays were performed in triplicate, and *P* value less than 0.05 (marked with an asterisk, ∗) was considered statistically significant.

We next examined if REM inhibits nuclear translocation of YB-1, a key transcription factor regulating *MDR1* gene expression. MCF-7/Dox cells were treated with LEM or REM for 6 hours and then examined a localization of endogenous YB-1 in the cells. Although YB-1 was diffusely found in the cells, it mostly localized in the nucleus. While LEM treatment did not affect YB-1 localization pattern, REM disrupted nuclear localization of YB-1 (Figure 3(a)). 

Accordingly, to examine if REM inhibits YB-1 interaction with MDR1 promoter, we performed the chromatin immunoprecipitation assays with the anti-YB-1 antibody. Our data from the chromatin immunoprecipitation assays showed that REM but not LEM inhibited YB-1 binding onto MDR1 promoter region (Figure 3(b)), which indicates that REM inhibits YB-1 interaction with MDR1 promoter. 

Thus, we further examined if REM effect was mediated by inhibiting transcriptional activity of YB-1. When MCF-7/Dox cells were cotransfected with MDR1-luc and YB-1 and then treated with REM for 6 hours, REM treatment reduced YB-1-induced luciferase activity (Figure 3(c)). 

### 3.4. REM-Activated JNK1/2 Inhibits YB-1-Dependent MDR1 Expression

**Figure 4:**
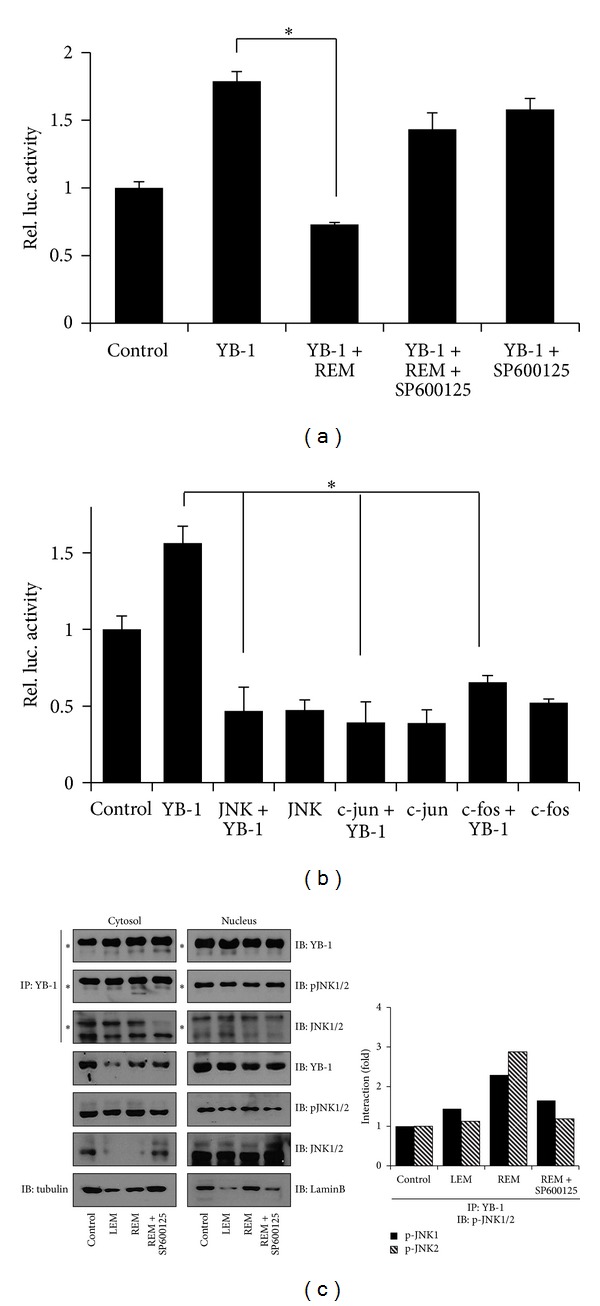
REM activation of JNK inhibits YB-1-dependent MDR1 expression. (a-b) MCF-7/Dox cells were transfected with MDR1-luc and constructs indicated for 24 hours. REM ± SP600125 was treated for 6 hours. Assays were performed in triplicate, and *P* value less than 0.05 (marked with an asterisk, ∗) was considered statistically significant. (c) YB-1 interaction with pJNK1/2. Cytosolic and nuclear proteins were immunoprecipitated with anti-YB-1 antibody. The immunoprecipitants and input proteins were then blotted with the antibodies for pJNK1/2, YB-1, JNK1, and actin. Asterisks indicate heavy chains from the immunoprecipitation. Quantitative analyses of protein interactions were performed using the Image J software.

Thus, we examined whether JNK1/2 inhibition rescues REM reduction of YB-1-dependent MDR1 expression. In the luciferase assays, SP600125 blocked REM inhibition of YB-1-dependent MDR1 promoter activity (Figure 4(a)). Thus, we further examined whether JNK1/2 signaling to c-Jun/c-Fos mediates REM inhibition of YB-1-dependent MDR1 expression. In the luciferase assays, we found that overexpression of JNK1, c-Jun, or c-Fos significantly reduces basal and YB-1-dependent MDR1 promoter activity (Figure 4(b)). 

So, we further examined whether JNK1/2 directly inhibits YB-1 transcriptional activity by repressing YB-1 nuclear translocation. In our immunoprecipitation assays with anti-YB-1 antibody, we found that REM causes pJNK1/2 interaction with YB-1 in the cytosol and a reduction of YB-1 level in the nucleus, while YB-1 is distributed in both the cytosol and nucleus of the untreated cells (Figure 4(c)). Thus, our data indicate that REM-induced JNK1/2 activation may lead to malfunction of YB-1 through a direct interaction in the cytosol.

### 3.5. REM-Activated JNK1/2 Reduces Viabilities of Multidrug- Resistant Cells

**Figure 5:**
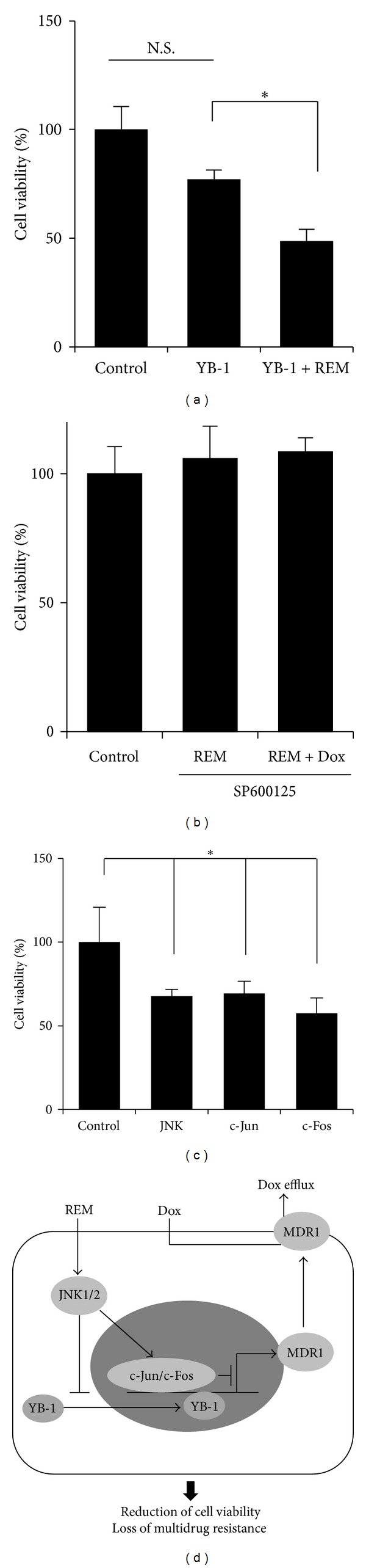
REM-induced JNK reduces MCF-7/Dox cell viability. (a) Cells were transfected with YB-1 and treated with REM 48 hours. The MTT experiments were performed in triplicate. N.S. indicates no significance in statistics. ∗, *P* < 0.05. (b) Cells were pretreated with SP600125 for 30 minutes and then treated with REM alone or REM plus doxorubicin for another 48 hours. The MTT assays were done in triplicate. (c) Cells were transfected with the indicatives for 48 hours and then subjected to the MTT assays. The experiments were performed in triplicate. ∗, *P* < 0.05. (d) Schematic illustration. YB-1-dependent MDR1 expression results in multidrug resistance by drug efflux (herein, doxorubicin). REM treatment activates JNK1/2, which blocks YB-1 nuclear translocation and induces c-Jun/c-Fos inhibition of YB-1-dependent MDR1 expression. Thus, REM results in the decrease of multidrug-resistant cancer cell viability with a loss of multidrug-resistant phenotype.

Our serial data hypothesize that REM-activated JNK1/2 inhibition of YB-1-dependent MDR1 expression may result in the reduction of cell viability. Thus, we further examined whether REM inhibits the viability of cells overexpressing YB-1. When cells were transfected with YB-1 and treated with REM for 48 hours, YB-1 overexpression itself did not significantly alter cell viability. However, REM reduced the viability of cells overexpressing YB-1 by approximately 61% (Figure 5(a)). 

So, we next examined whether JNK1/2 inhibition rescued REM reduction of cell viability. When cells were pretreated with SP600125 for 30 minutes and then treated with REM for another 48 hours, REM did not affect cell viability. Furthermore, a combination of REM with doxorubicin also did not affect the viability of the cells pretreated with SP600125 (Figure 5(b)). 

As REM activation of JNK1/2 reduced MDR1 expression level, we further examined whether JNK1/2 affects cell viability. When MCF-7/Dox cells were transiently transfected with JNK1, c-Jun, or c-Fos and then subjected to the MTT assays, the overexpression of JNK, c-Jun, or c-Fos reduced the viabilities by approximately 30% to 50% (Figure 5(c)). 

## 4. Discussion

Multidrug resistance of cancer cells results in poor prognoses. MDR1 expression upon a treatment of chemotherapeutic agents gains that phenotype [5, 8, 31, 32]. In this study, we provide knowledge that REM, the extract from white mulberry roots, reduces the viabilities of multidrug-resistant MCF-7/Dox cells by inhibiting YB-1-dependent MDR1 expression via JNK1/2 activation. 

Inhibitions of drug efflux function of MDR1 have been issued in treatment of multidrug-resistant cancer cells expressing MDR1. In our study, REM reduced both MCF-7 and MCF-7/Dox cells, which were enhanced by doxorubicin addition. This additive effect is likely due to REM reduction of MDR1 expression, as this reduced MDR1 level causing doxorubicin accumulation in the cells (Figure 5(d)). Furthermore, REM-induced JNK1/2 pathway caused the reduction of MDR1 expression and cell viability, which was rescued by SP600125 but not by SB203580, PD98059, and LY294002 (data not shown). Consistently, we found that MCF-7/Dox cell viability was reduced by overexpression of JNK1, c-Jun, or c-Fos. Recently, it has been found that MDR1 silencing with MDR1 shRNA reduces multidrug-resistant tumor cell proliferation [21], which indicates that a direct inhibition of MDR1 expression could be another option for treating multidrug-resistant cancer cells. Thus, the activation of JNK1/2 pathway to inhibit MDR1 expression is likely to be another option for treating multidrug-resistant cancer cells. In our preliminary cytotoxicity studies, multidrug-resistant leukemic cells were also sensitive to REM. Furthermore, REM repressed YB-1 transcriptional activity for MDR1 expression in those cell types (data not shown). Accordingly, REM but not LEM may affect viabilities of different types of drug-resistant cancer cells. Meanwhile, REM protected doxorubicin-induced cell death of H9c2 cardiac myoblast cells [33]. Thus, it is likely that REM effectively kill drug-resistant cancer cells with no or less side effect of doxorubicin *in vivo*. 

JNK1/2 has been shown to negatively regulate different types of multidrug-resistant cancer cells such as multidrug-resistant EPG85-257RDB gastric cancer cells and EPP85-181RDB pancreatic cancer cells [17]. JNK1/2 activity was also negatively correlated with MDR1 expression in hepatocarcinoma cell lines [34]. Consistently, photosensitizer pheophorbide a based photodynamic therapy induced the apoptosis multidrug-resistant R-HepG2 cells via JNK1/2 activation [35]. Similarly, JNK1/2 activation by PSC833, a cyclosporine analogue, inhibited MDR1 expression in doxorubicin-resistant SK-MES-1/DX1000 lung cancer cell line [36]. It was recently shown that JNK1/2 involves DKK-3 in the apoptosis of MCF-7/Dox cells [37]. In addition, JNK1/2-mediated c-Jun activation inhibited MDR1 expression in multidrug-resistant K562/A02 cells [38]. Furthermore, c-Fos inhibited MDR1 expression in MCF-7 cells [20]. Thus, our data that REM-induced JNK1/2 inhibits MDR1 expression and multidrug-resistant cell viability is relevant to recent findings. However, JNK1/2 mediated hypoxia-induced MDR1 expression in HeLa cells [39]. Likewise, COX2-mediated JNK1/2-c-Jun activation appears to contribute multidrug resistance of HCT8/V colorectal cancer cells [40]. Therefore, it is likely that JNK1/2 regulation of multidrug-resistant cancer cells is dependent on mechanisms acquiring multidrug-resistant phenotype. Thus, the effect of REM remains to be tested in particular mechanisms such as hypoxia-induced MDR1 expression. Nevertheless, our findings here strongly suggest that REM regulation of JNK1/2 inhibits YB-1-dependent MDR1 expression in multidrug-resistant cancer cells. 

In this study, we first provide evidence that REM activation of JNK1/2 reduces multidrug-resistant cancer cells by targeting YB-1-dependent MDR1 expression. Nevertheless, remains functions of REM against multidrug-resistant cancer cells in the *in vivo* experiments to be verified. Traditional medicines have long been used, suggesting that prescriptions based on traditional medicines work well in particular disease conditions. However, we still do not know what chemical components in REM uniquely play roles for JNK1/2 inhibition of YB-1-dependent MDR1 expression in multidrug-resistant cancer cells. Thus, it is required to understand the exact biochemical and molecular mechanisms by which REM works against multidrug-resistant cancer cells. That effort will improve the quality of traditional medicines with view of biomedical sciences.

## 5. Conclusion

This study demonstrates that REM but not LEM causes the reduction of multidrug-resistant cancer cell viability by JNK inhibition of YB-1-dependent MDR1 expression. Although we still explore to find the active chemical compounds that make the unique REM function against multidrug-resistant cancer cells, our study is expected to provide knowledge for REM effect against cancer disease.
